# Left Pulmonary Artery Stenting for Left Pulmonary Artery Stenosis Following Patent Ductus Arteriosus Device Closure: Case Series and Review of the Literature

**DOI:** 10.1155/2024/6690515

**Published:** 2024-06-24

**Authors:** Víctor Molina, Mehdi Hadid, Joaquim Miró, Nagib Dahdah

**Affiliations:** ^1^ Division of Pediatric Cardiology Centre Hospitalier Universitaire Sainte-Justine University of Montreal, Montreal, Canada; ^2^ Cardiology Department Dr. Luis Calvo Mackenna Hospital, Santiago, Chile

## Abstract

Percutaneous device occlusion is currently the standard of care for most cases of patent ductus arteriosus (PDA). Albeit infrequent, device-related left pulmonary artery (LPA) stenosis is a known complication of this procedure, occasionally requiring stent placement to relieve the obstruction. We present a series of four patients who required left pulmonary stenting after ductus arteriosus device closure. A review of the current evidence is presented.

## 1. Introduction

Percutaneous closure of the patent ductus arteriosus (PDA) was first introduced by Porstmann et al. in the late 1960s [1, 2]. Since then, technical advances and the development of new devices have made it the standard treatment for PDA occlusion. The standard technique for device PDA closure requires a protrusion of the coil or the device into the main pulmonary artery (MPA). The anatomical proximity of the left pulmonary artery (LPA) to the PDA puts it at risk of device-related obstruction, a potential complication that has been long known since the early experiences with the Rashkind Ductal Occluder® [3], with Gianturco coils [4], and with the Amplatzer ductal occluder® (ADO) [5], this last one being the most widely used device to this date. This complication of the procedure is becoming more frequent as smaller patients are benefiting from the intervention, in whom the PDA-adjacent structures are proportionally smaller than the size of the PDA and of the device [6]. Most PDA device-related LPA stenoses improve with time and do not need intervention [6]. However, occasional patients will potentially require stenting of the LPA. We present a series of four patients in whom PDA device-related LPA stenosis occurred and in which stenting of the LPA was performed.

## 2. Case 1

A 1-year-old girl was diagnosed with a medium-sized PDA and mild LPA stenosis. Left atrial and left ventricle enlargement was noted on transthoracic echocardiography (TTE). The PDA was described as possibly having an unusual shape, so a cardiac scan was done which showed a regular-shaped 3.4-mm-diameter PDA at the pulmonary side. An LPA proximal stenosis was also observed and measured at 4.3 mm, with a distal LPA diameter of 7.4 mm. She was referred to the catheterization laboratory for PDA occlusion. A type-A PDA was identified by angiography, with a diameter of 3.3 mm at the pulmonary side and 7.4 mm at the aortic ampulla. The Qp/Qs was 1.5, and the pulmonary vascular resistance (PVR) was normal. A 12 mmHg systolic pressure gradient was measured between the MPA and the LPA. The PDA was easily closed with an ADO 10–8 device (Abbott Structural Heart, Plymouth, MN, USA). No residual shunt was observed. Postprocedure echocardiography showed no residual shunt and the same LPA stenosis anatomy with no gradient at Doppler interrogation.

**Figure 1:**
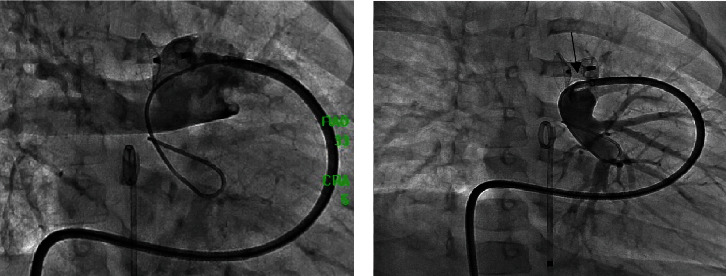
(a) Pulmonary artery angiography showing no pass of contrast to the LPA. (b) Selective injection in the proximal LPA shows a tight stenosis (arrow) adjacent to the PDA-device.

**Figure 2:**
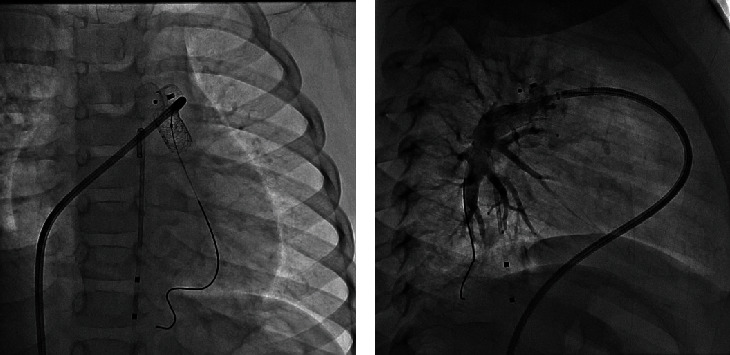
(a) Correct position of proximal LPA stent and (b) LPA contrast injection in straight lateral projection showing good angiographic result.

At follow-up, she continued to be asymptomatic, but progressive LPA stenosis was noted on echocardiography. At 3 years of age, a lung perfusion scintigraphy showed a 25% perfusion to the left lung and 75% to the right lung, and a cardiac scan showed a 3 mm proximal LPA. She was then taken to the catheterization laboratory for LPA stenting. Angiography showed a proximal LPA distortion in relation to the device with a 2.8 mm proximal LPA diameter that increased to 6.1 mm distally (Figure 1). A 12-mmHg MPA–LPA gradient was measured with normal right ventricle (RV) pressure. An 8 × 20 mm Formula-418 biliary stent (Cook Medical, Bloomington, IN, USA) was deployed over an 8 mm balloon. The final stent diameter was 7.3 mm (Figure 2). Postprocedure echocardiography showed no gradient at the LPA level, and lung perfusion scintigraphy the next day showed a 31% perfusion to the left lung. She was discharged the next day. Follow-up has been uneventful, and a redilatation of the stent after somatic growth is foreseen.

## 3. Case 2

A full-term newborn girl with a birth weight of 3.5 kg was diagnosed at 15 days of age with a large 5.3-mm PDA. Left chamber dilatation was noted, and the pulmonary arteries were of normal size. She was hospitalized at 28 days of age due to congestive heart failure and inadequate weight gain despite diuretics, digital treatment, and nasogastric tube feeding. At 5 weeks of age, with a weight of 3.7 kg, she was referred to the catheterization laboratory for PDA closure. During the diagnostic phase of the procedure, she had a short episode of ventricular fibrillation attributed to wire manipulation in the LV, which was properly reanimated and cardioverted. A type-C 4.5-mm PDA was observed with a length of 7.3 mm. PDA closure with an 8 mm Amplatzer Vascular Plug II (AVP II, Abbott Structural Heart, Plymouth, MN, USA) was attempted multiple times from the pulmonary side, but the device was not stable, and it easily went through the PDA when trying to deploy, so it was finally withdrawn. Finally, a 10-mm AVP II device was adequately deployed, showing stability and no residual shunt. A device-related LPA stenosis was noted the next day on echocardiography, and a lung perfusion scintigraphy showed a 12% perfusion to the left lung. She was discharged 7 days after the procedure.

**Figure 3:**
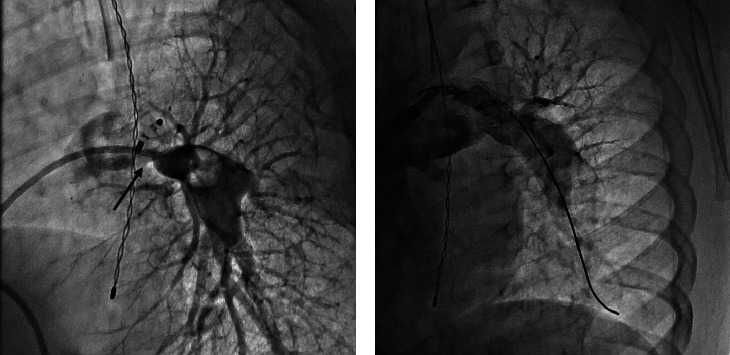
(a) LPA angiography in straight lateral projection shows severe device related LPA stenosis (arrow). (b) LPA angiography in LAO projection after stenting shows the stent in good position and a good angiographic result.

At follow-up, cardiac failure symptoms had subsided, and diuretics were stopped. She remained asymptomatic, but LPA stenosis continued to be noted by echocardiography, with a 15–25 mmHg peak gradient. At 30 months of age, a lung perfusion scintigraphy was performed, which showed a 13% perfusion to the left lung. She was sent to the catheterization laboratory for LPA stenting. The diagnostic study showed a mildly hypertensive RV (31/8 mmHg; 40% systemic) and severe LPA stenosis. Distal LPA pressure was measured at 11/8 mmHg (close to nonpulsatile), with an MPA–LPA gradient of 19 mmHg. Angiography showed a 2-mm proximal LPA stenosis in contiguity to the device. An 8 × 16 mm Formula-418 biliary stent (Cook Medical, Bloomington, IN, USA) was deployed. The angiographic result was optimal, RV pressure was 25/8 mmHg postprocedure, and the postprocedural left lung perfusion increased to 38% (Figure 3). A mild dysphonia and stridor were noted after the procedure, and a recurrent laryngeal nerve compression was considered a possibility. However, symptoms were mild, so no further tests were considered necessary at that moment. At follow-up, 1 week after, no stridor or dysphonia were noted. At her last follow-up, 4 months after the procedure, she was still doing well, and no MPA–LPA gradient was noted by echocardiography. A stent dilatation is scheduled to be done after somatic growth.

## 4. Case 3

A 2-month-old full-term girl was referred to the cardiology clinic due to failure to thrive and a heart murmur. Her weight was 3.4 kg, and mild heart failure symptoms were noted. A large 4-mm PDA was diagnosed, and left atrium and ventricle dilation were noted. Five weeks later, the PDA was still the same size, and her weight was 4.3 kg. She was referred to the catheterization laboratory for PDA closure due to a dilated left ventricle and elevated pulmonary artery pressure (estimated to be 80% systemic). The diagnostic study showed systemic PA pressure and a Qp/Qs of 1.8. A type-C PDA was diagnosed and measured 6 mm at the pulmonary end and 8 mm at the aortic ampulla. An 8-mm AVP-II was deployed but was considered unstable and therefore replaced by a 10-mm AVP-II. No residual shunt was observed. The device protruded mildly into the descending aorta, and a 4 mmHg gradient between the aortic arch and the descending aorta was measured. At postprocedure echocardiography, the pulmonary-side disk of the device was shown to obstruct the LPA, with a proximal diameter of 2 mm and a 22-mmHg peak gradient. Pulmonary scintigraphy showed a 35% perfusion to the left lung, so no intervention was considered necessary at that moment. At 2 weeks of follow-up, symptoms had subsided and weight gain had improved. At 6 months, the proximal LPA stenosis measured 2 mm with a peak gradient of 48 mmHg. At 18 months old, she was clinically fine, but flow to the LPA was no longer visible by echocardiography, and lung perfusion scintigraphy yielded 2% perfusion to the left lung. She was referred to the catheterization laboratory for LPA stenting.

**Figure 4:**
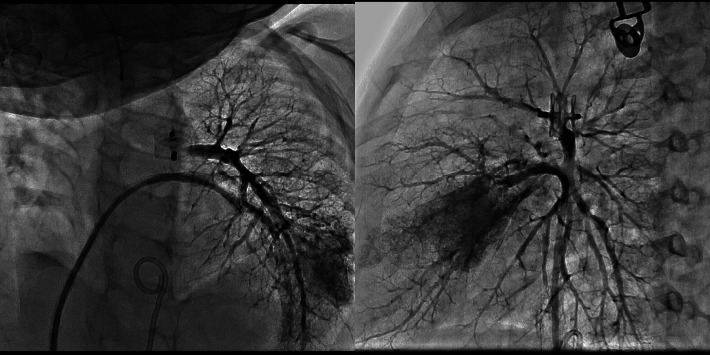
Wedged angiography in left upper pulmonary vein (lingular branch) showing the anatomy of the LPA distal to the obstruction. No reflux to the MPA is seen due to complete obstruction of the LPA.

**Figure 5:**
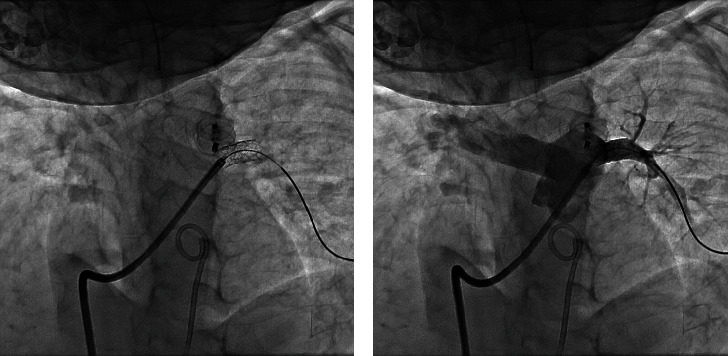
Final (a) stent position and (b) MPA angiography in cranial-LAO projection showing unobstructed LPA after stenting.

The diagnostic study showed a complete occlusion of the LPA. No gradient was observed at the aortic isthmus level. Wedged angiography in the left lower pulmonary vein showed that the LPA was patent and measured 4 mm at the hilum level, but that it was obstructed by the device at its ostium (Figure 4). The obstruction was crossed successfully with a 0.035^″^ straight-tipped Terumo Glidewire (Terumo Medical Corporation, Somerset, NJ, USA), and subsequently, a 5 × 16 mm Formula-418 biliary stent was deployed (Figure 5). No procedure-related complications were observed. Echocardiography showed the stent in a good position with no residual gradient. No complications occurred. Heparin perfusion was administered until the next day, and aspirin was prescribed afterwards. The follow-up was uneventful. Pulmonary scintigraphy showed left lung perfusion of 14% at 1 day, 29% at 1 month, and 31% at 5 months after the procedure. At her last follow-up at 4 years of age, she was doing well, and on echocardiography, the flow was present to the LPA with an MPA–LPA peak gradient of 15 mmHg. The decision was made to continue treatment with aspirin due to the small size of the stent and LPA-occlusion history. A dilatation of the stent is expected following somatic growth.

## 5. Case 4

An 8-month-old girl was referred for PDA device closure in the context of symptoms of heart failure and left chamber dilation. Diagnostic catheterization showed a Qp/Qs of 2.3 and a type-A PDA with a minimal diameter of 2.5 mm. The PDA was closed with an ADO 6/4 device. She was followed clinically and by serial echocardiography and lung perfusion scintigraphy. A somewhat stable LPA stenosis was observed, and at 11 years of age, left pulmonary perfusion was 34%. It was decided to repeat a lung perfusion scintigraphy after somatic growth. At 15 years of age, a 5.1-mm LPA stenosis with a 15-mmHg peak gradient was observed at echocardiography, and left lung perfusion was 24%, so an LPA stenting was decided.

The diagnostic study showed a 6.1-mm proximal LPA stenosis with a 15.5-mm diameter at the level of the hilum. The MPA–LPA systolic gradient was 16 mmHg, and RV pressure was near normal (33% systemic). A balloon interrogation of the lesion with a 16 *mm* × 3 *cm* Z-Med balloon (Numed for Children, Orlando, FL, USA) showed an elastic lesion that left a 14.7-mm waist at the level of the stenosis (in relation to the device). An 8-zig 28-mm CP stent (Numed for Children, Orlando, FL, USA) mounted on a 16 *mm* × 3 *cm* BIB balloon (Numed for Children, Orlando, FL, USA) was initially used; however, during deployment, the stent migrated proximally into the MPA. The stent was then apposed to the MPA vascular wall with further expansion with a 25 *mm* × 4 *cm* Tyshak balloon (Numed for Children, Orlando, FL, USA), followed by a 28 *mm* × 4 *cm* Z-Med balloon (Numed for Children, Orlando, FL, USA). The stent was successfully stabilized in the MPA, but severe pulmonary regurgitation was observed at angiography and at echocardiography due to stent-related pulmonary valve entrapment. The procedure was stopped to avoid further migration of the stent, and a decision was made to postpone the LPA stenting for another procedure.

**Figure 6:**
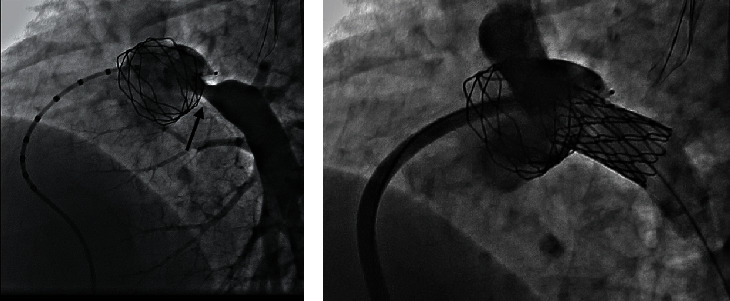
Proximal LPA stenosis is noted on angiography (arrow). (a) The stent in the MPA is also seen. (b) Good angiographic result after LPA stenting.

Free pulmonary regurgitation was clinically well tolerated. A treadmill exercise test 1 year after the procedure was normal (14.3 min duration, 17.5 METS). Slow progression of RV dilatation was observed, and cardiac MRI obtained 2 years after the procedure showed a fractional pulmonary regurgitation of 27% and a left lung perfusion of 23%. RV was of normal size, and the RV and left ventricle ejection fraction were normal. One year later, a lung perfusion scintigraphy showed a left lung perfusion of 19%. After a discussion with the family, it was decided to attempt an LPA stenting again. At 18 years of age, she was brought to the catheterization laboratory. A proximal 7.1-mm LPA stenosis was noted. The LPA was successfully stented with a 28-mm CP stent mounted on a 16 *mm* × 3 *cm* BIB balloon (Numed for Children, Orlando, FL, USA). The stent was well placed, and its final diameter at the level of the stenosis was 15.5 mm (Figure 6). Postcatheterization pulmonary scintigraphy showed a 32% perfusion to the left lung. A mild stridor was noted immediately after the procedure, and there was some dysphonia afterwards. A recurrent laryngeal nerve compression was suspected, but since the symptoms were mild, no further evaluation was done at the moment. Follow-up was uneventful, and dysphonia progressively resolved. A percutaneous pulmonary valve implantation is foreseen in case of significant RV dilatation.

## 6. Discussion

Percutaneous device occlusion is currently the standard of care for most PDA cases. Several different devices have been historically used. Beyond the neonatal period, the ADO is the most commonly used device for large PDAs. Albeit infrequent, device-related LPA obstruction is one of the known possible complications of this procedure and has been described with almost every known device [7, 8].

The true frequency of this complication is not clearly known since there are no acknowledged diagnostic criteria for PDA-device-related LPA stenosis. Although the complication can be easily confirmed in severe cases, this might not be the case in moderate or mild cases. In the latter situations, redistribution of flow to the right lung usually diverts blood flow from the left lung and hence leads to underestimating the actual pressure gradient at the level of the left-side stenosis. Device protrusion over the LPA emergence is common with different devices, and it can happen in over 90% of cases with the use of coils, but its occurrence does not necessarily predict a left lung perfusion abnormality [9, 10]. However, even if the presence of device protrusion into the MPA does not predict an LPA obstruction, it is logical to think that it is a prerequisite to developing this complication. An abnormal postdevice placement left lung perfusion has been described in 16%–37% of cases with different devices [11–15]. All of these reports consider left lung perfusion below 40% as abnormal [11–15]. The timing of the perfusion study could be relevant since an obstruction might not be initially present but may develop progressively, as described in premature born babies [16]. Also, left lung perfusion can get better with time [14, 15]. Kramoh et al. [14] studied a large consecutive series of PDA coil occlusion with lung perfusion scans immediately after intervention with a mid-term follow-up. Normalization was observed in around 67% of coil occlusion cases at a mean of 20 months of follow-up, and further normalization was seen in 50% of the remaining abnormal perfusion patients at a mean of 40 months of follow-up. Patients who received an ADO showed improvement in left lung perfusion without normalization, but this group was small (*n* = 10) for any definitive statistical conclusion. Since there have been no subsequent studies based on device implantation, the possibility of an underdiagnosis of this complication should be kept in mind. Nevertheless, it remains unclear whether mild to moderate LPA stenosis needs to be addressed.

Some risk factors for post-PDA device LPA stenosis have been identified: young age [11–14], low weight [11, 13, 14], larger PDA size for body surface area [14], and ductal length [11, 13]. There is a growing body of evidence in neonates and specifically in premature babies ever since the Piccolo device (Abbott Structural Heart, Plymouth, MN, USA) was formally introduced to the market, suggesting that small infants and particularly preterm babies are at higher risk of LPA obstruction following device placement. In a study by Chien et al., 57% of premature born babies of less than 2500 g had an LPA obstruction at different follow-up stages [16]. It is worth noting that Cases 2 and 3 of our series were 1 month old and 3 months old, respectively, and that they were less than 4.5 kg at the moment of the procedure. Both had a large type-C (tubular) PDA (4.5 mm and 6 mm, respectively) and required the use of a double-disk device (AVP-II). A disk protruding in the MPA would, at least in theory, increase the risk of LPA obstruction. Indeed, in a multicentric study regarding PDA closure with AVP-II and ADO II AS devices, LPA stenosis was observed in 4.5% of patients with the use of AVP-II, which increased to 12.5% of patients with a weight of less than 5 kg. In this series, LPA stenosis was usually mild, and only 2 of 19 patients required surgical removal of the device for this reason [17]. As previously stated, there is no clear relation between device protrusion to the LPA and impaired left lung perfusion. Intriguingly, no clear correlation exists between flow velocities measured by echocardiography or pressure pull-back measurements and lung perfusion alterations (11–13). However, a Doppler Velocity Index [(*LPA* *velocity*–*RPA* *velocity*)/*pulmonary* *trunk* *velocity* × 100] of 50% or more has been shown to be a good predictor of abnormal LPA perfusion after PDA device closure [9]. Similarly, a baseline MPA–LPA invasive gradient of >7 mmHg can be predictive of patients who would progress towards a postprocedural LPA stenosis defined as a left lung perfusion of <35% [18].

Overall, it seems clear that the PDA closure device can protrude into the LPA in some patients and cause left lung perfusion abnormalities. However, the clinical impact of the perfusion abnormalities and the cut-off perfusion level at which an intervention might be encouraged are not clear. Normal lung perfusion distribution in adults is around 52% to the right lung and 48% to the left lung [19], and the usually accepted normal distribution is 55% to the right lung and 45% to the left lung [14, 20]. Of course, these are average values and do not take into account the perfusion variations that could theoretically occur in the presence of a longstanding PDA. The absence of symptoms is falsely reassuring since patients with unilateral pulmonary stenosis are usually asymptomatic and underestimate their degree of exercise limitation as measured by exercise testing [21]. In a study by Hiremath et al., patients with unilateral pulmonary stenosis had a median pulmonary blood flow maldistribution (PBFM, i.e., percentage points from the normal 55%–45% distribution) of 19.5% and showed decreased exercise capacity and ventilatory inefficiency, as shown by a decreased peak VO2 and a high V_E_/VCO2, respectively, the latter as a result of V/Q mismatch [21]. Moreover, pulmonary artery stenting resulted in an improvement in peak VO2 and V_E_/VCO2, probably relating to improvement of V/Q mismatch, stroke volume response during exercise, and pulmonary regurgitation [21]. However, this was studied mainly in patients having complex corrected congenital heart disease, such as tetralogy of Fallot, D-transposition of great arteries, and double outlet RV. In addition, 50% of the cohort had at least moderate pulmonary insufficiency, a condition that can worsen in the presence of unilateral pulmonary artery stenosis [22, 23].

A 2011 statement from the American Heart Association for pediatric cardiac catheterization recommended pulmonary branch angioplasty when there is a relative flow discrepancy between the 2 lungs of 35%/65% or worse [24]. Hiremath et al. suggested an algorithm for decision-making in unilateral pulmonary stenosis that essentially states that intervention should be carried out when symptoms are present, peak-to-peak gradient is >20 mmHg at the level of the obstruction, or lung perfusion is >10% (percentage points) from normal, that is, <35% to the left lung or <45% to the right lung [20]. Once again, these recommendations are mainly for patients with complex heart defects who might get a greater benefit from a low threshold for intervention. The threshold at which LPA stenting might be beneficial in isolated PDA patients is less clear and should be weighed against the potential risks of stenting. In our institution, LPA stenting is usually considered when left lung perfusion is <25% (and RPA stenting is considered when right lung perfusion is <30%), but it is also considered in cases of left lung perfusion of 25%–30% or right lung perfusion of 30%–35% in the context of complex heart defects with significant pulmonary regurgitation.

Pulmonary artery stenting is a well-established treatment for pulmonary artery stenosis. Given that significant LPA stenosis after PDA device closure is rare and often asymptomatic, LPA stenting is infrequent but can be mandatory in severe cases, as in Case 3 of our series. The 4 cases that we present come from a total cohort of 206 cases of PDA device closure in the same period. This constitutes a 2% risk of needing LPA stenting after PDA device closure in our experience. We should note, however, that 2 of those 4 cases were patients less than 3 months old with a large PDA and in which an AVP-II device was used. LPA stenting in this setting was reported for the first time in 2013 [25], and a few other cases have been reported in the literature [26], with good results. Although stenting is a relatively common procedure in the congenital catheterization laboratory, it is not without risks. Potential complications are well known and will not be discussed here, but they can be significant, as in Case 4 of our series. A particular complication of LPA stenting in the presence of a PDA device is the occurrence of vocal cord paralysis due to impingement of the left recurrent laryngeal nerve between the device and the LPA stent [27]. In a retrospective study regarding vocal cord paralysis after LPA stenting and/or PDA device closure, involving 5364 patients in 12 institutions, this complication was seen in 2/4001 cases of PDA device closure, in 6/1337 cases of LPA stenting, and in 4/26 cases of PDA device closure + LPA stenting (15%), with most of the cases recovering completely [28]. In our series, this complication was suspected in two patients, but symptoms were mild and transitory, so no further tests or particular management was considered necessary.

## 7. Conclusion

Device closure is currently the standard treatment for PDAs. The refining of the technique and of the material has made it a very safe procedure. However, complications are still possible and more frequent as progressively smaller patients are intervened. Among these, postprocedure LPA stenosis is a rare but potentially significant long-term complication. Accurate diagnosis and severity assessment of this condition is difficult, but lung perfusion scintigraphy seems to be the best modality to evaluate the physiological impact of the obstruction. Cases of significant obstruction are rare but can be treated with LPA stenting. The left lung perfusion cut-off level that merits an intervention is not clear, as the benefit in mild or moderate cases is debatable in the absence of significant congenital heart disease. Potential risks of stenting still exist, and vocal cord paralysis, although apparently transitory, should be clearly discussed while obtaining consent. As such, the decision to intervene must be tailored to the individual patient.
